# Supplementary material for: Resolving out of Africa event for Papua New Guinean population using neural network
Source: Nat Commun. 2025 Jul 9;16:6345. doi: 10.1038/s41467-025-61661-w (PMC12241555; doi:10.1038/s41467-025-61661-w)
Supplement: Supplementary file 1 — Supplementary Information [file 41467_2025_61661_MOESM1_ESM.pdf]

Resolving out of Africa event for Papua New Guinean population using neural network

Mayukh Mondal[1,2*], Mathilde André[2,3,4], Ajai K. Pathak[3,5], Nicolas Brucato[6], François-Xavier Ricaut[6], Mait Metspalu[3], Anders Eriksson[2]

Affiliations:

1. Institute of Clinical Molecular Biology, Christian-Albrechts-Universität zu Kiel 24118 Kiel, Germany.
2. Centre for Genomics, Evolution & Medicine, Institute of Genomics, University of Tartu, Riia 23b, 51010 Tartu, Tartumaa, Estonia.
3. Estonian Biocentre, Institute of Genomics, University of Tartu, Riia 23b, 51010 Tartu, Tartumaa, Estonia.
4. Dept. of Neurosciences "Rita Levi Montalcini", University of Turin, Italy.
5. Department of Human Genetics, KU Leuven, 3000 Leuven, Belgium.
6. Centre de Recherche sur la Biodiversité et l'Environnement (CRBE), Université de Toulouse, CNRS, IRD, Toulouse INP, Université Toulouse 3 – Paul Sabatier (UT3), Toulouse, France.

* Corresponding author: mondal.mayukh@gmail.com

Supplementary Figures

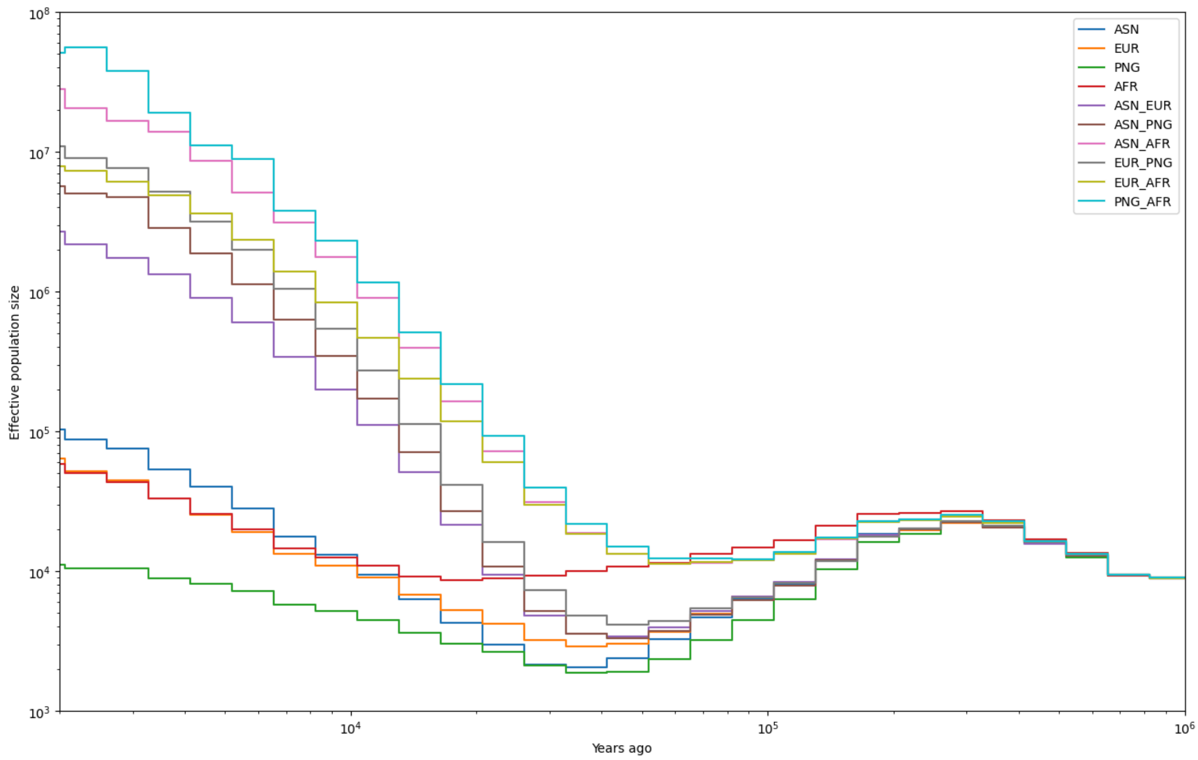

Supplementary Figure 1: Relate results of effective population size on empirical data using 40 samples per population. AFR = Yoruba, EUR = British from England and Scotland, ASN = Han Chinese and PNG = Highlanders of Papua New Guinea. The x-axis is years ago from modern times with the log scale. The y-axis is effective population size also in the log scale.

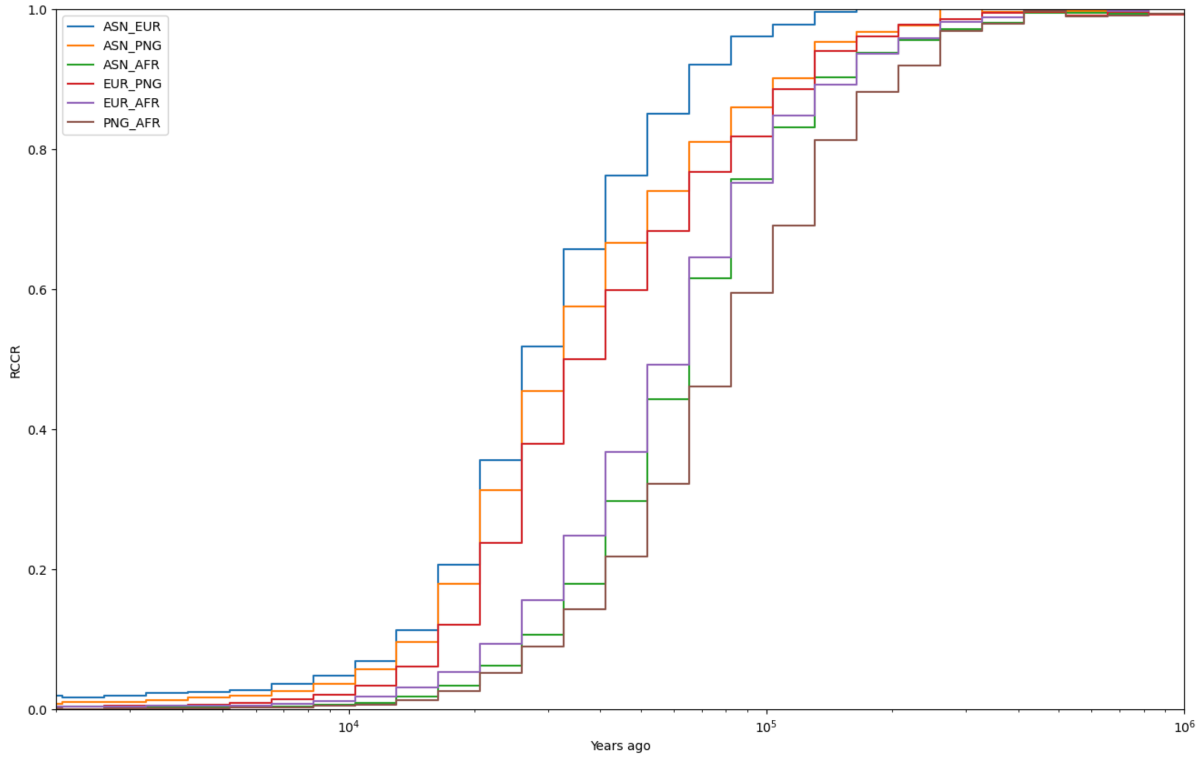

Supplementary Figure 2: Relate results of relative cross coalescent rate (RCCR) curve on empirical data using 40 samples per population. AFR = Yoruba, EUR = British from England and Scotland, ASN = Han Chinese and PNG = Lowlanders of Papua New Guinea. The x-axis is years ago from modern times with the log scale. The y-axis is RCCR values calculated by Relate.

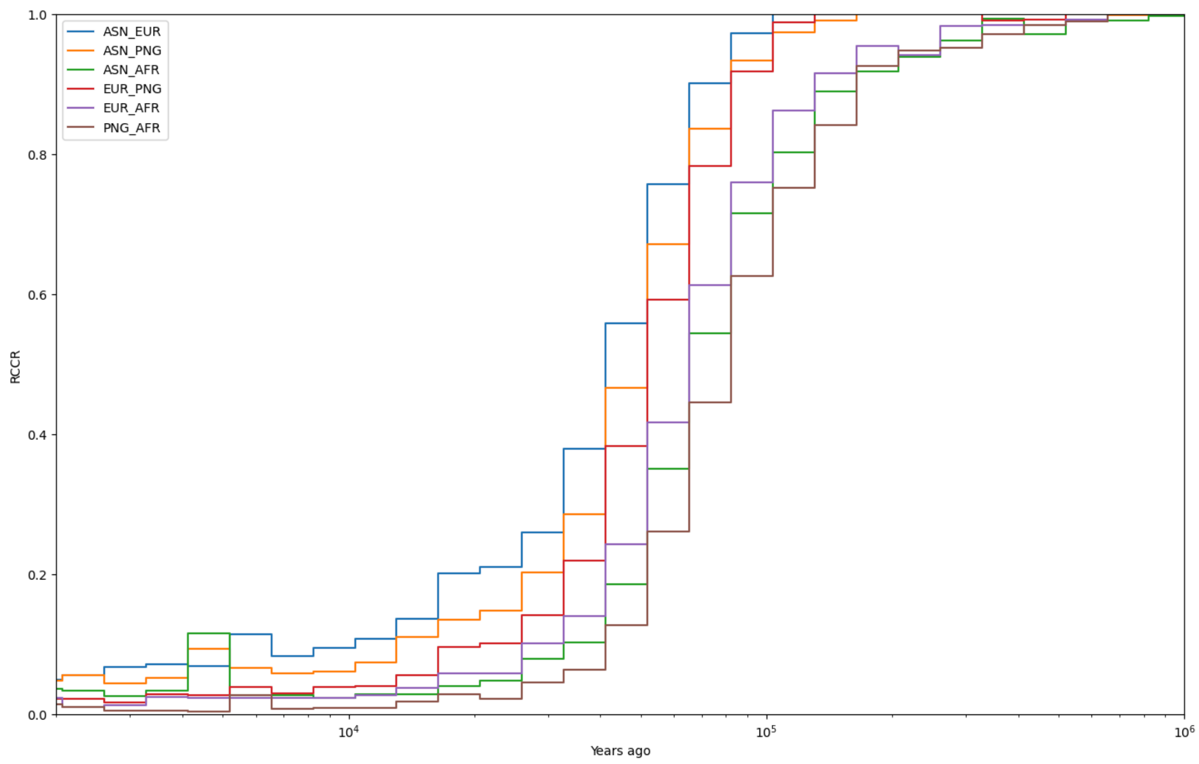

Supplementary Figure 3: Relate results of relative cross coalescent rate (RCCR) curve on physically mapped samples using two samples per population. AFR=Yoruba, EUR=Sardinian, ASN=Han and PNG=Highlands of Papua New Guinea. The x-axis is years ago from modern times with the log scale. The y-axis is RCCR values calculated by Relate.

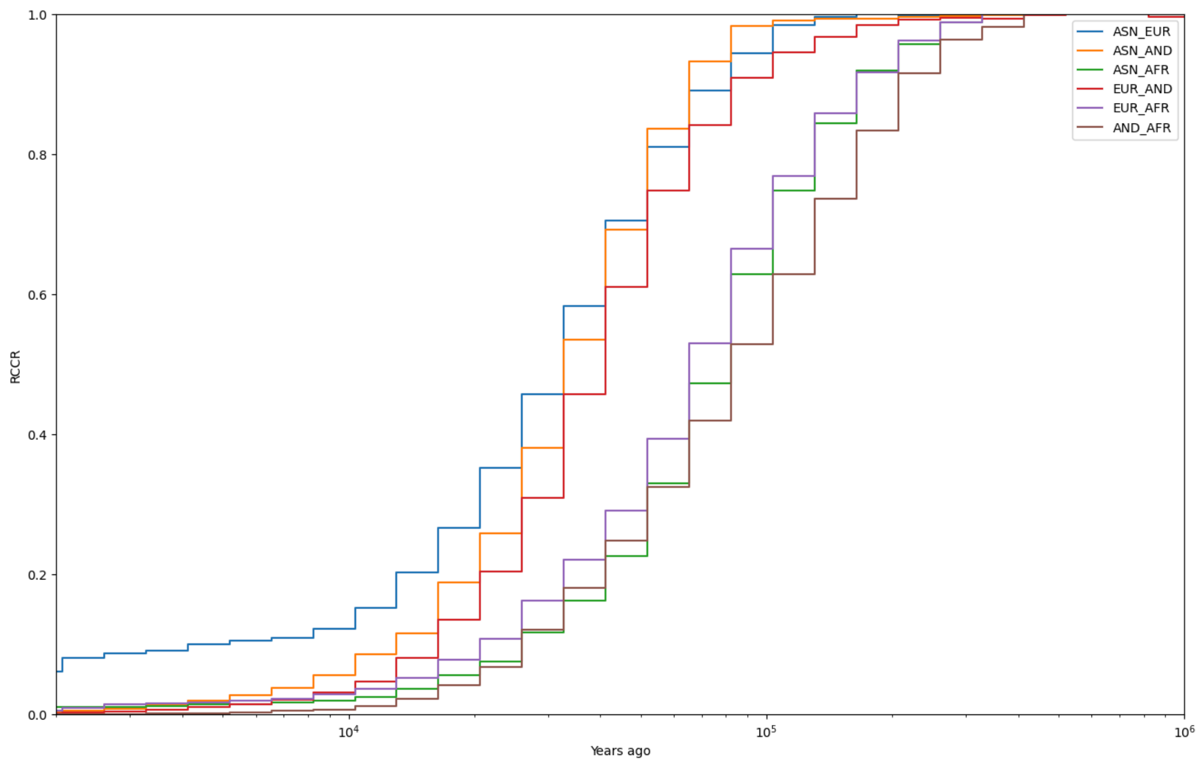

Supplementary Figure 4: Relate results of relative cross coalescent rate (RCCR) curve on empirical data using 10 samples per population. AFR = Yoruba, EUR = British from England and Scotland, ASN = Han Chinese and AND=Andamanese. The x-axis is years ago from modern times with the log scale. The y-axis is RCCR values calculated by Relate.

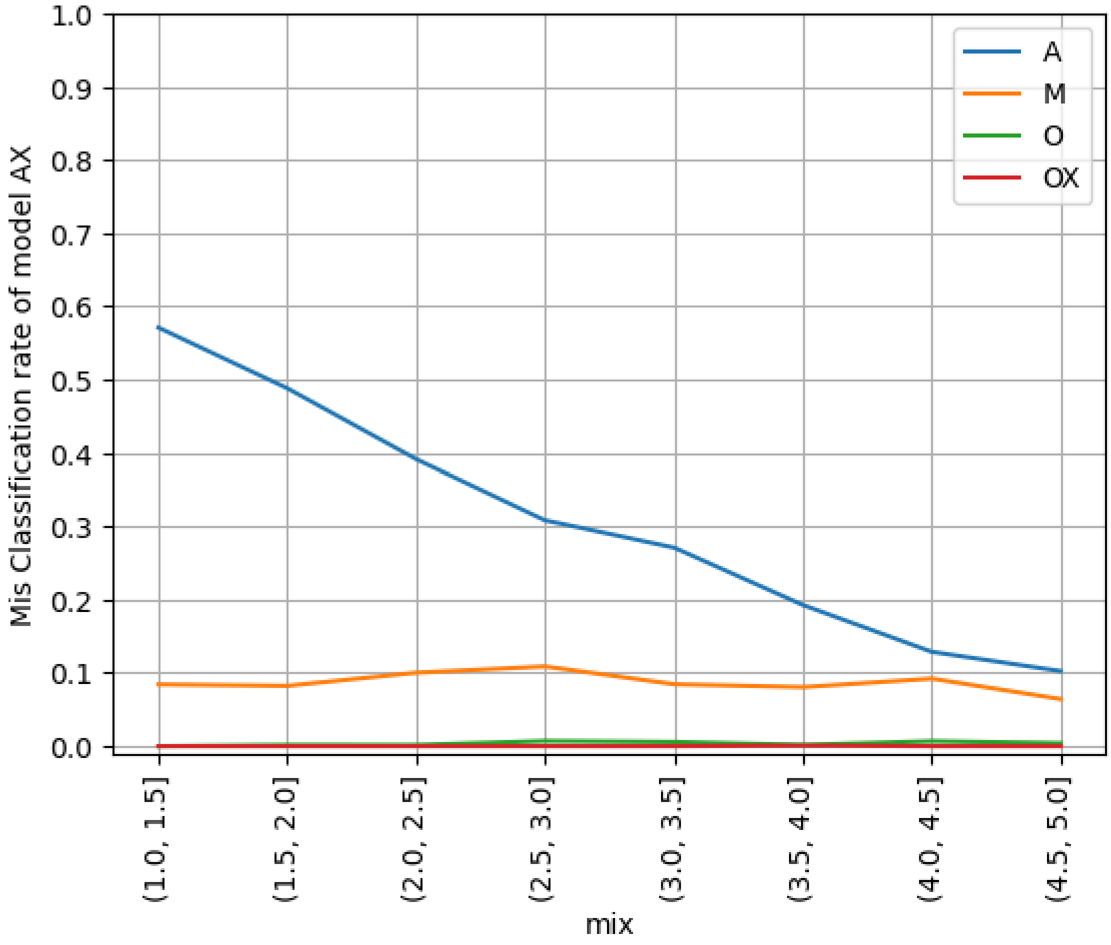

Supplementary Figure 5: Misclassification rate of ABC-DLS for Model AX to other model for admixture level less than 5% from earlier OOA population. The misclassification was calculated when ABC-DLS predicted the wrong model with a bayes factor more than 10.

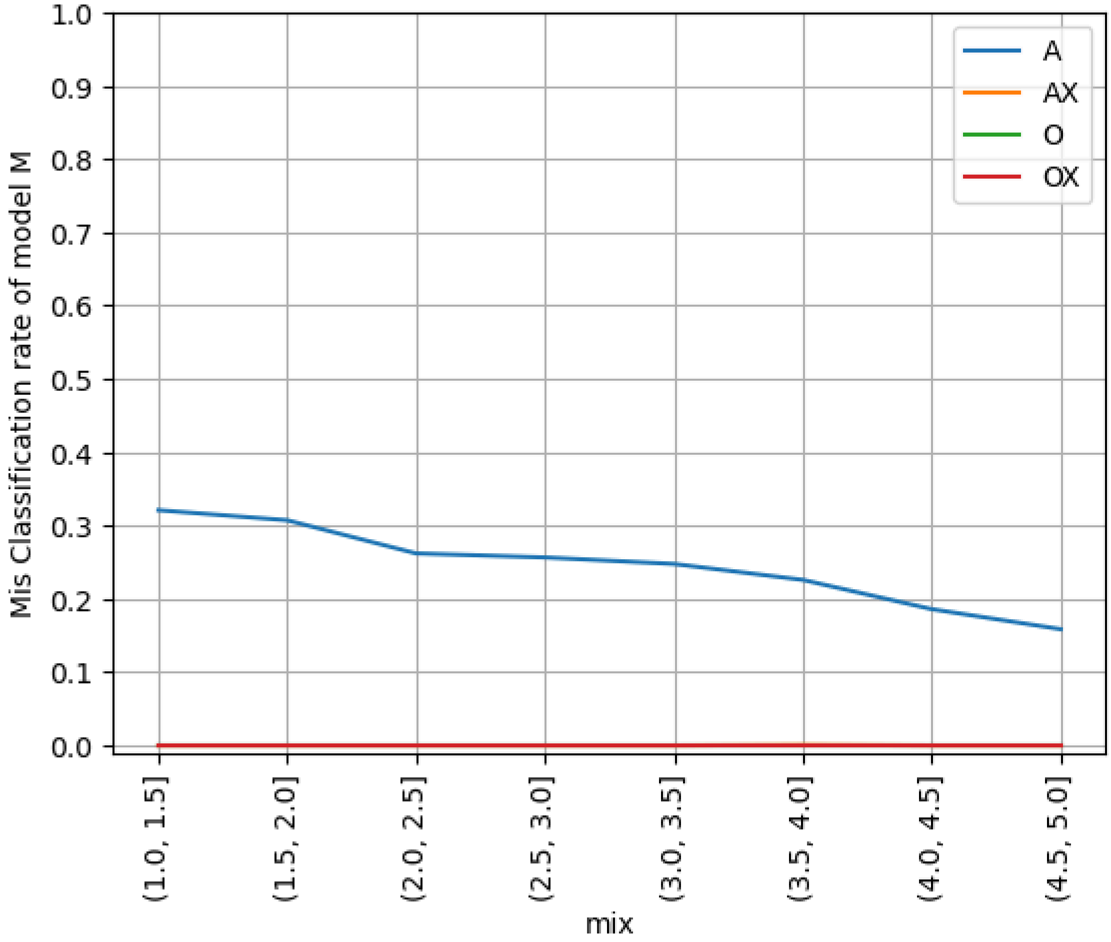

Supplementary Figure 6: Misclassification rate of ABC-DLS for Model M to other model for admixture level less than 5% from an outgroup of European and East Asian. The misclassification was calculated when ABC-DLS predicted the wrong model with a bayes factor more than 10.

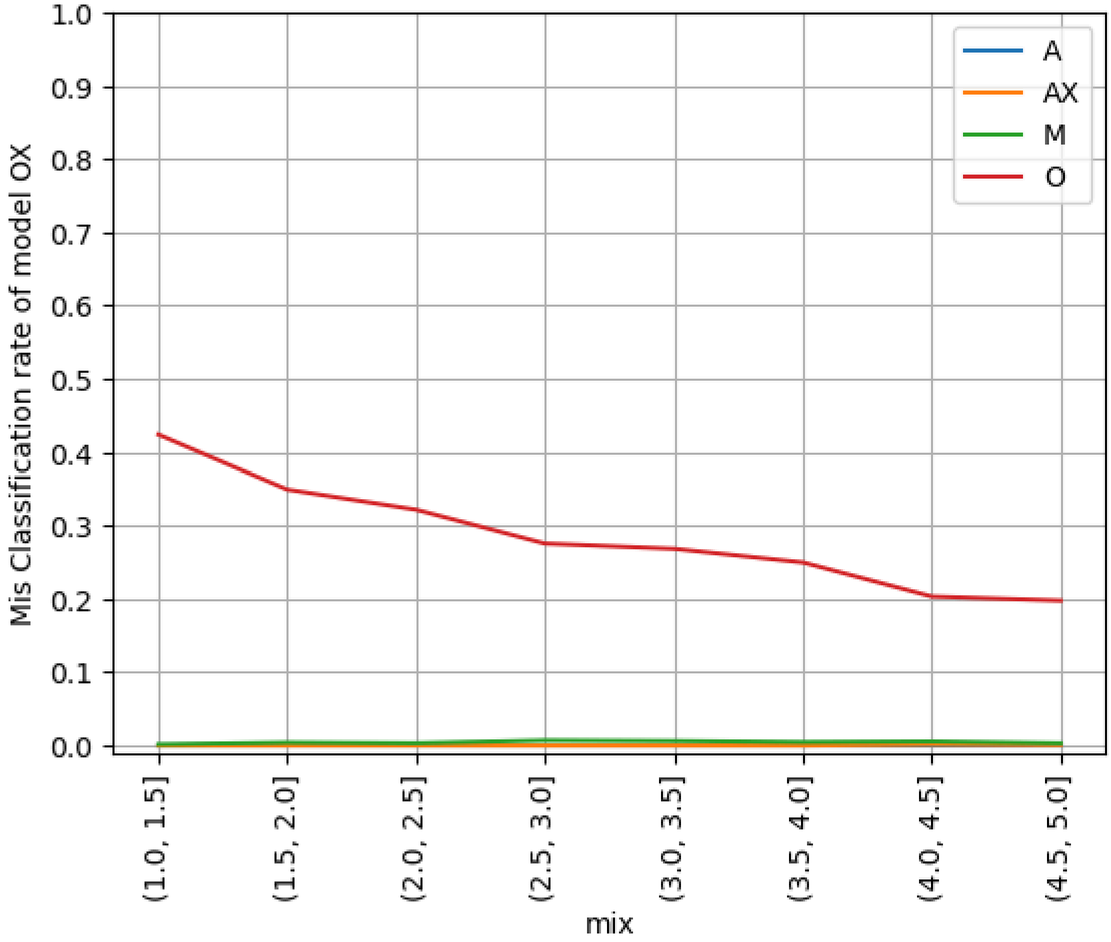

Supplementary Figure 7: Misclassification rate of ABC-DLS for Model OX to other model for admixture level less than 5% from earlier OOA population. The misclassification was calculated when ABC-DLS predicted the wrong model with a bayes factor more than 10.

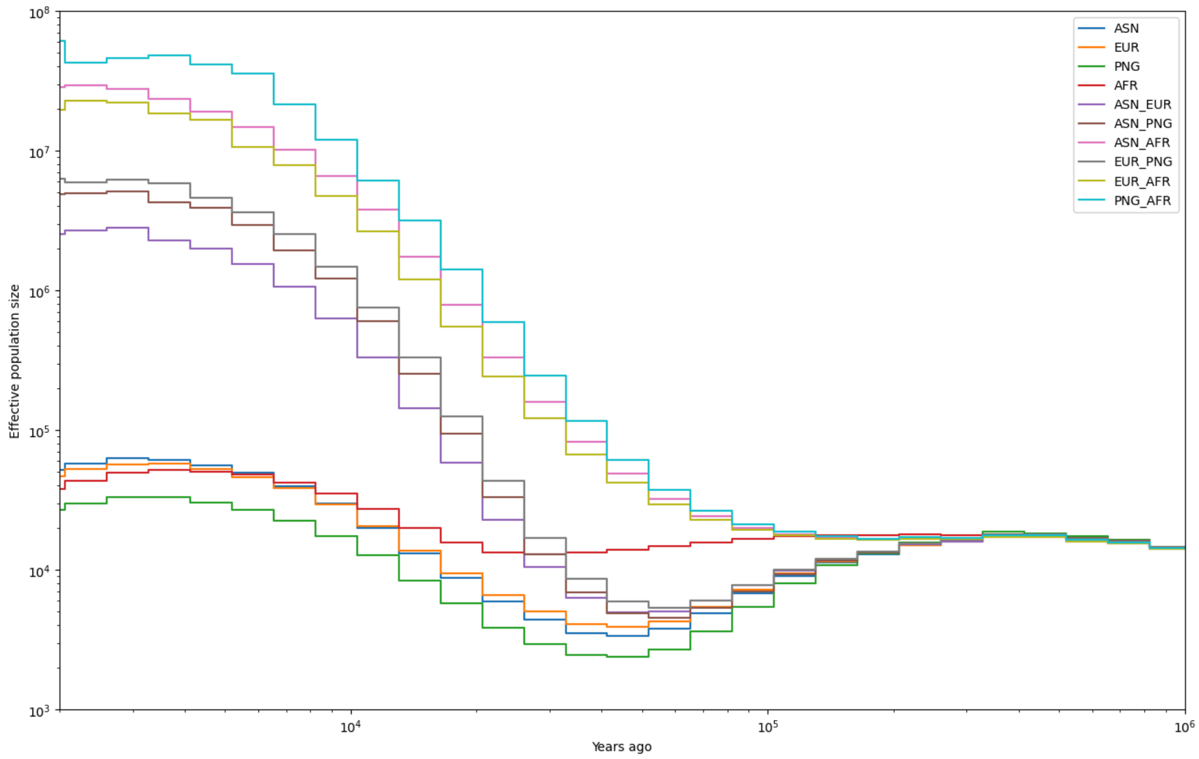

Supplementary Figure 8: Relate results of effective population size on simulated data of best fitted model A using 40 samples per population. AFR = Africa, EUR = European, ASN = East Asian and PNG = Papua New Guinea. The x-axis is years ago from modern times with the log scale. The y-axis is effective population size also in the log scale.

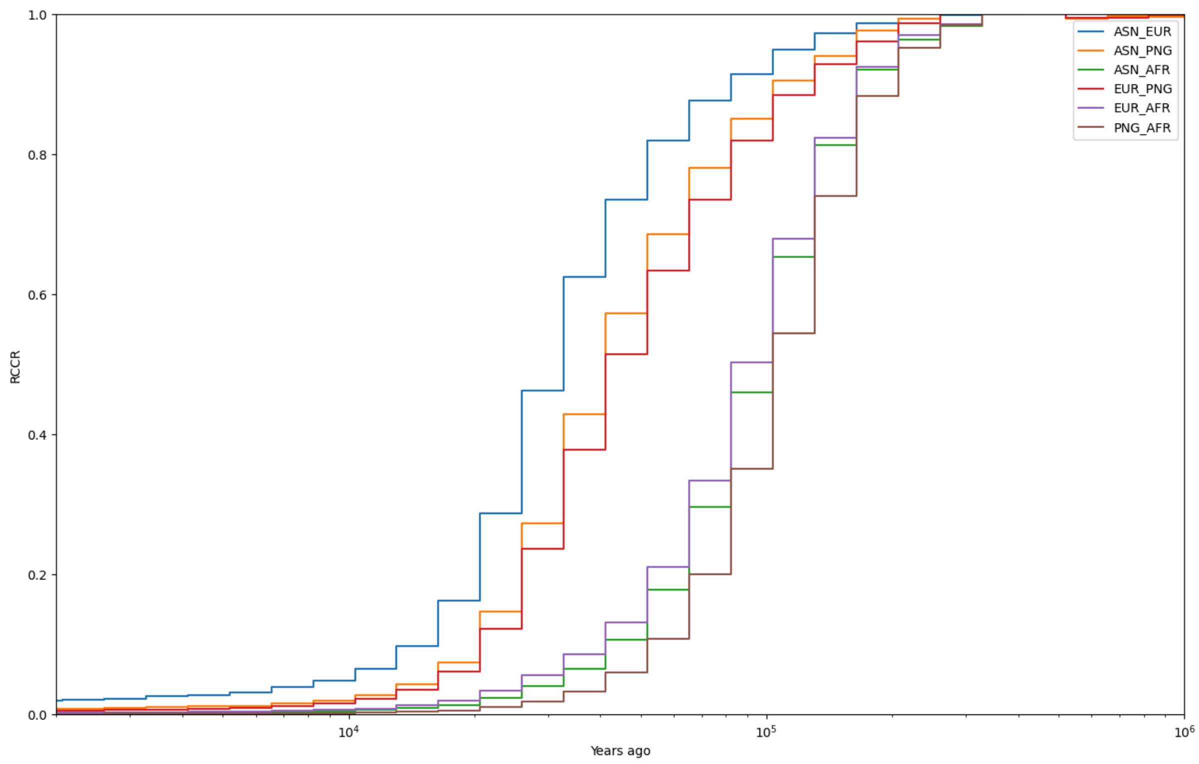

Supplementary Figure 9: Relate results of relative cross coalescent rate (RCCR) curve on simulated data of best fitted values from Model A without Archaic Introgression using 40 samples per population. AFR = African, EUR = European, ASN = East Asian and PNG = Papua New Guinean. The x-axis is years ago from modern times with the log scale. The y-axis is RCCR values calculated by Relate.

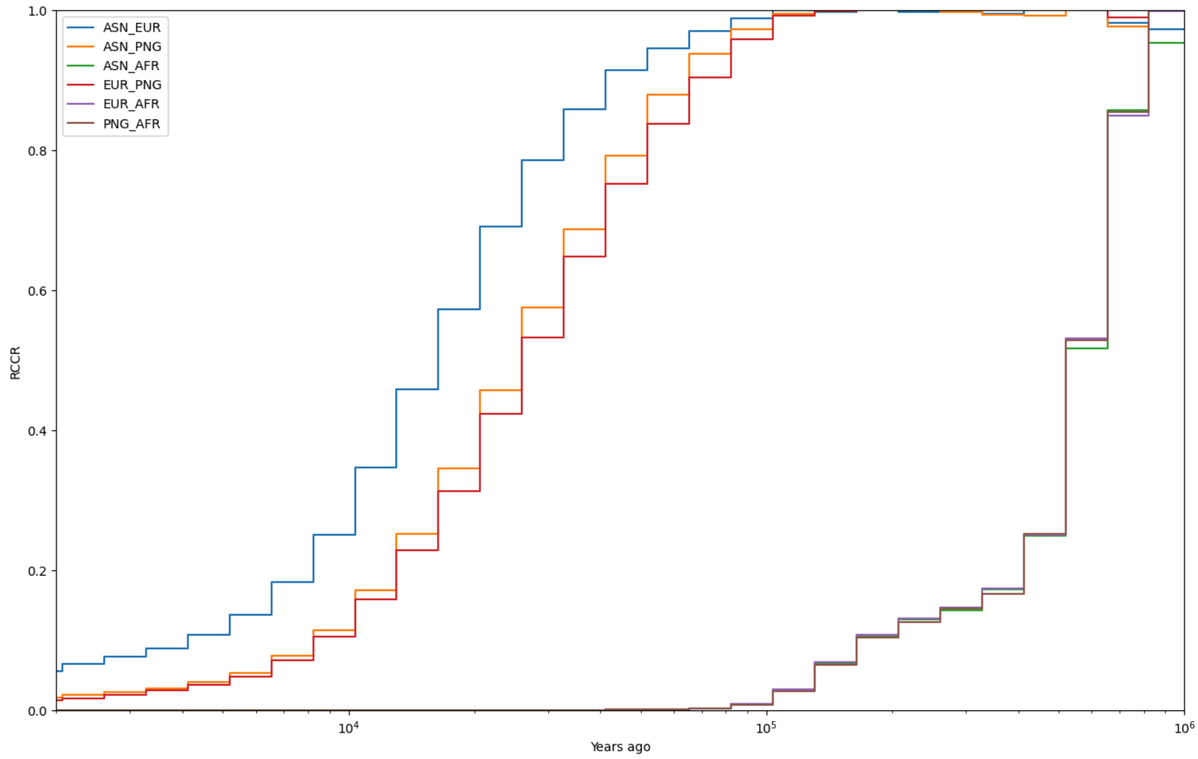

Supplementary Figure 10: Relate results of relative cross coalescent rate (RCCR) curve on simulated data of best fitted values from Model A with an African population separated 300 thousand years ago using 40 samples per population. AFR = African, EUR = European, ASN = East Asian and PNG = Papua New Guinean. The x-axis is years ago from modern times with the log scale. The y-axis is RCCR values calculated by Relate

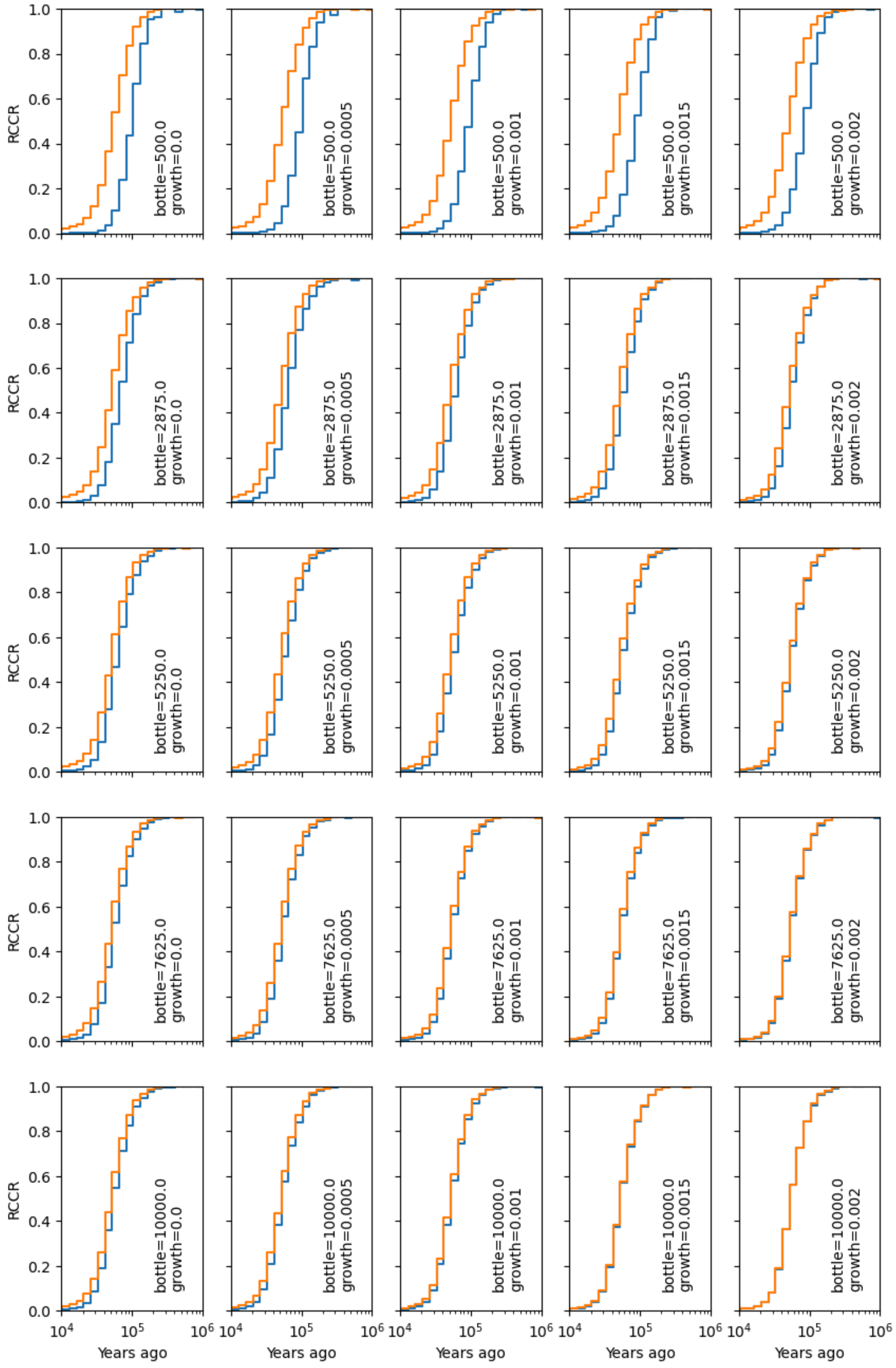
RCCR
Years ago
bottle=500.0 growth=0.0
bottle=500.0 growth=0.0005
bottle=500.0 growth=0.001
bottle=500.0 growth=0.0015
bottle=500.0 growth=0.002
bottle=2875.0 growth=0.0
bottle=2875.0 growth=0.0005
bottle=2875.0 growth=0.001
bottle=2875.0 growth=0.0015
bottle=2875.0 growth=0.002
bottle=5250.0 growth=0.0
bottle=5250.0 growth=0.0005
bottle=5250.0 growth=0.001
bottle=5250.0 growth=0.0015
bottle=5250.0 growth=0.002
bottle=7625.0 growth=0.0
bottle=7625.0 growth=0.0005
bottle=7625.0 growth=0.001
bottle=7625.0 growth=0.0015
bottle=7625.0 growth=0.002
bottle=10000.0 growth=0.0
bottle=10000.0 growth=0.0005
bottle=10000.0 growth=0.001
bottle=10000.0 growth=0.0015
bottle=10000.0 growth=0.002

Supplementary Figure 11: RCCR graph with combination of bottleneck and growth rate using simulated model using Minimal model of the out of Africa. Orange line is the Africa vs European RCCR curve which is constant throughout the graph, whereas the blue line is the Africa vs Papua New Guinean RCCR curve under different amount of bottleneck and growth rate (written inside the legend) for Papua New Guinean population.

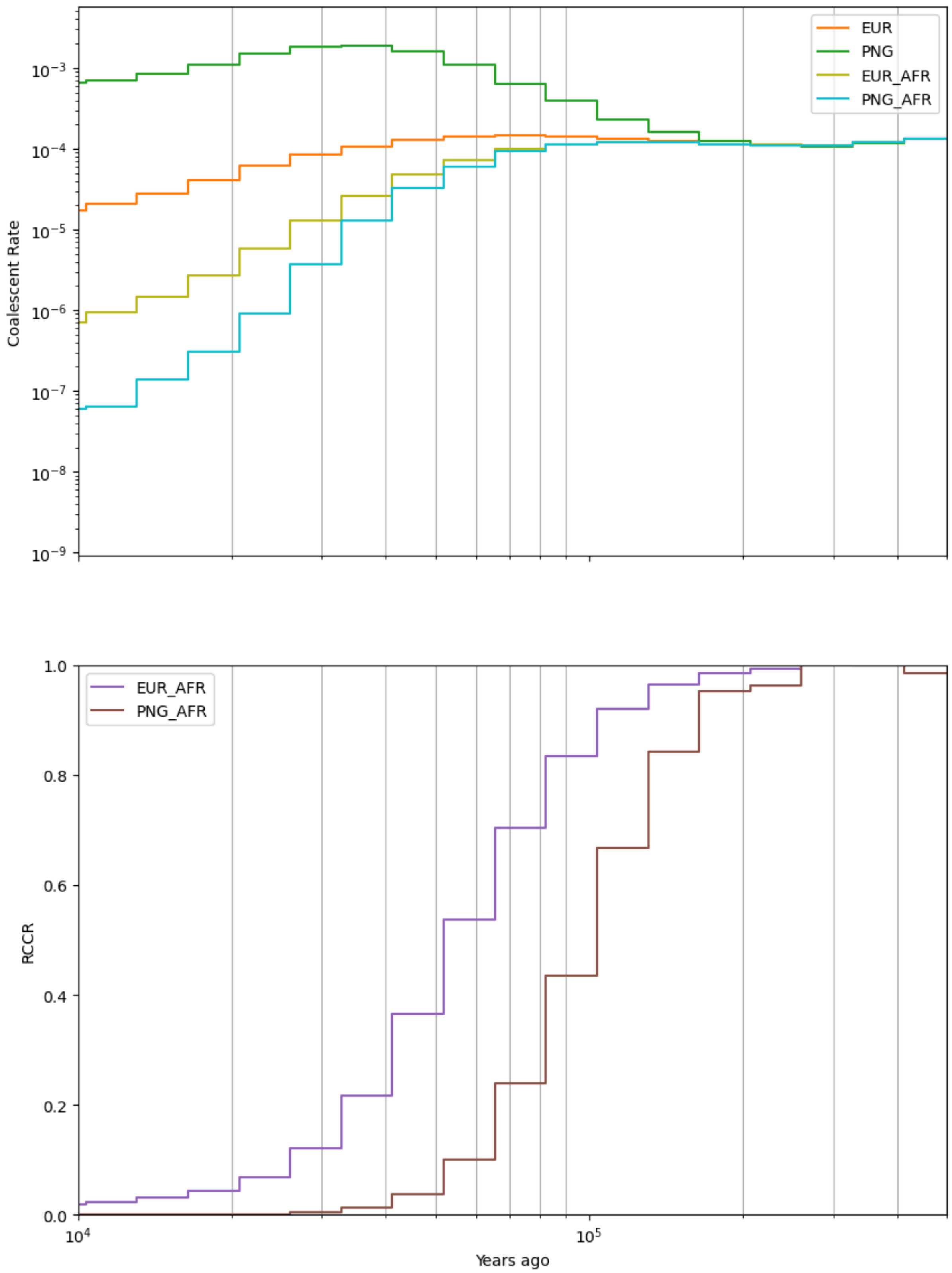
EUR
PNG
EUR_AFR
PNG_AFR
Coalescent Rate
10⁻³
10⁻⁴
10⁻⁵
10⁻⁶
10⁻⁷
10⁻⁸
10⁻⁹
EUR_AFR
PNG_AFR
RCCR
1.0
0.8
0.6
0.4
0.2
0.0
10⁴
10⁵
Years ago

Supplementary Figure 12: Coalescent rate and corresponding RCCR curve of a simple simulated model for the top separated RCCR curve between Africa vs European and Africa vs Papua New Guinean. The Papua New Guinean has a bottleneck of 500 individuals and growth rate 0. The European population never had a bottleneck and growth rate of 0.2%. EUR = European and PNG = Papua New Guinean.

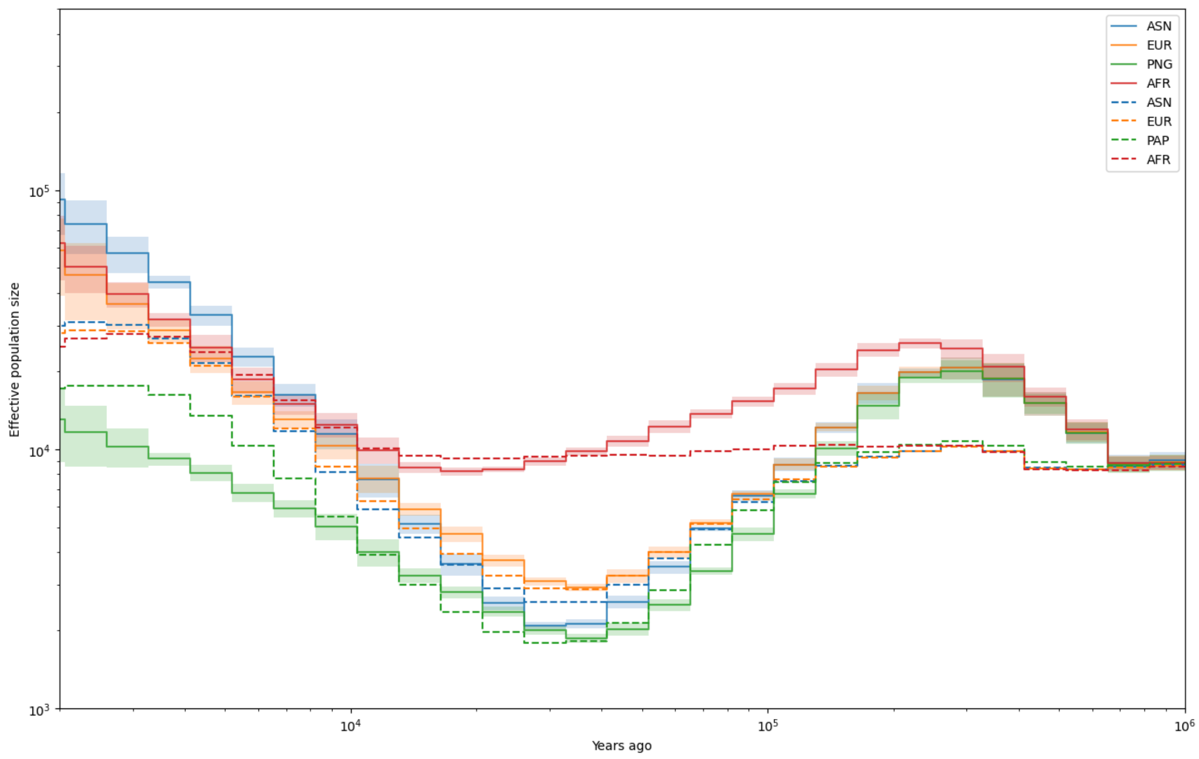

Supplementary Figure 13: Relate results of effective population size on empirical data using 40 samples per population. AFR = Yoruba, EUR = British from England and Scotland, ASN = Han Chinese and PNG = Highlanders of Papua New Guinea. The x-axis is years ago from modern times with the log scale. The y-axis is effective population size also in the log scale. The shaded regions are the 95 percent (two standard deviation) confidence intervals created from 10 separate random sampling events. The line represents the mean values. The dotted line represents the simulated data of best fitted model A.

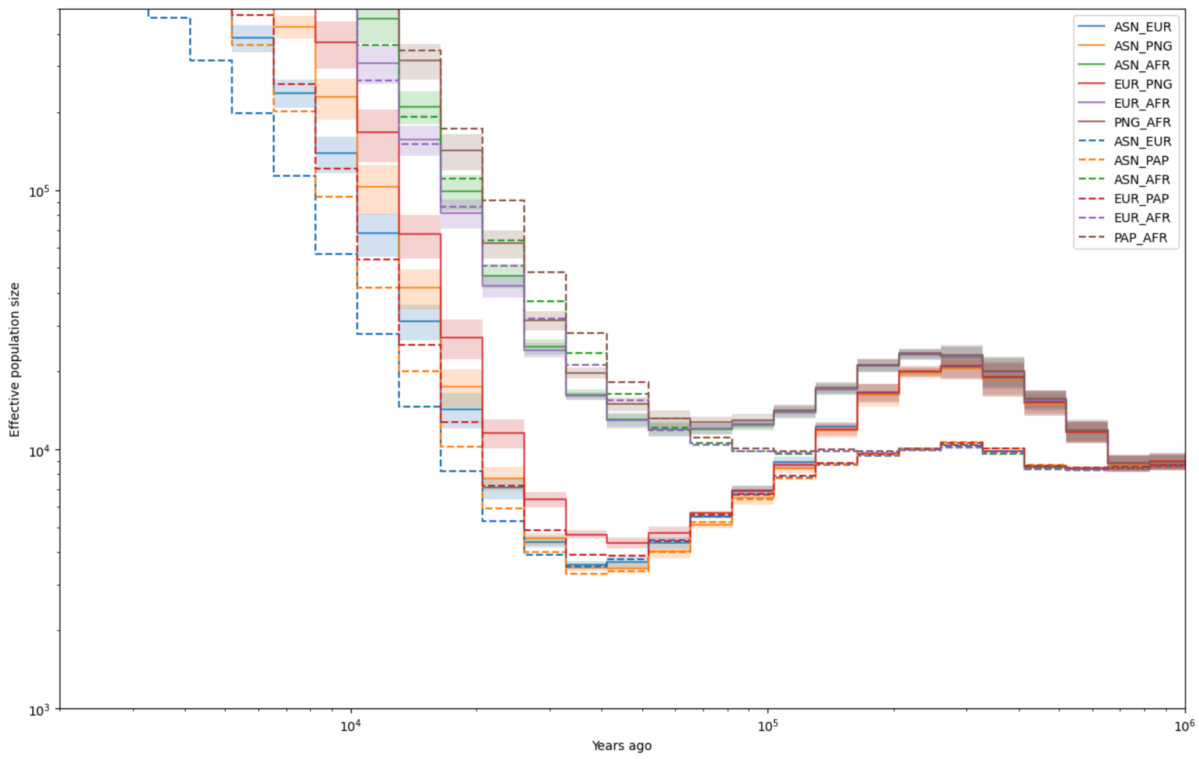

Supplementary Figure 14: Relate results of cross effective population size on empirical data using 40 samples per population. AFR = Yoruba, EUR = British from England and Scotland, ASN = Han Chinese and PNG = Highlanders of Papua New Guinea. The x-axis is years ago from modern times with the log scale. The y-axis is effective population size also in the log scale. The shaded regions are the 95 percent (two standard deviation) confidence intervals created from 10 separate random sampling events. The line represents the mean values. The dotted line represents the simulated data of best fitted model A.

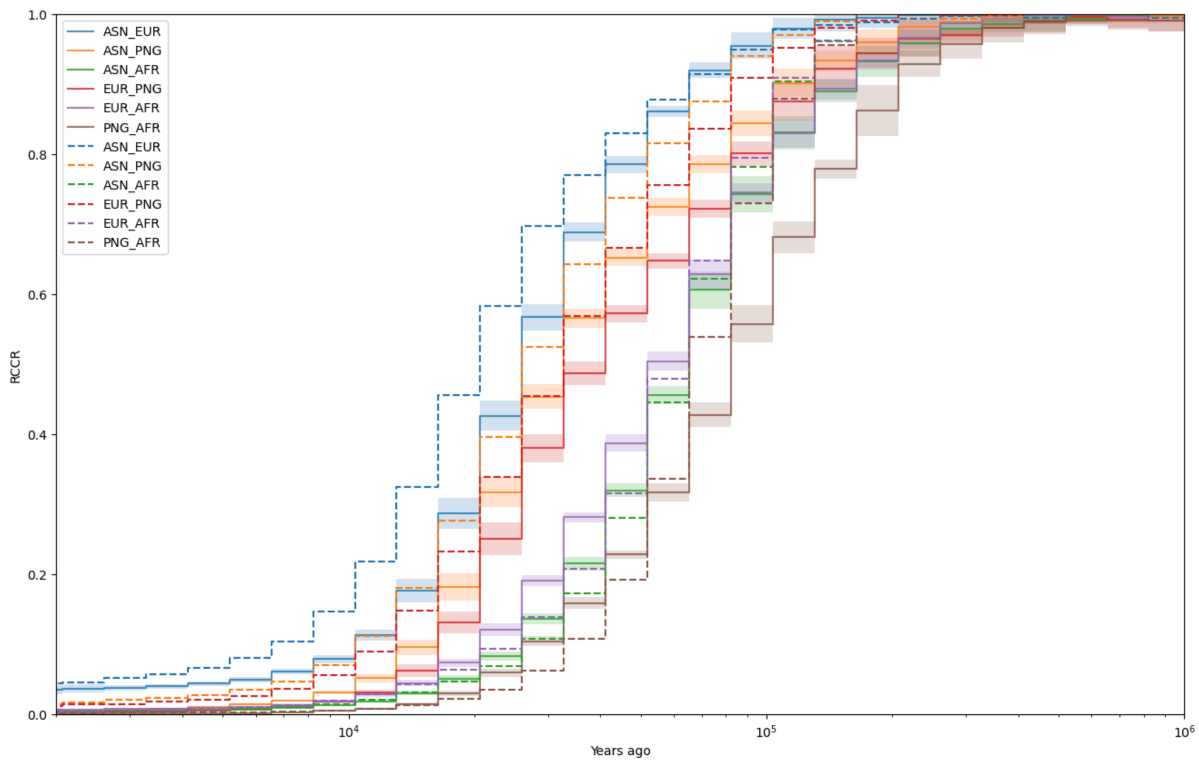

Supplementary Figure 15: Relate results of relative cross coalescent rate (RCCR) curve on empirical data using 40 samples per population. AFR = Yoruba, EUR = British from England and Scotland, ASN = Han Chinese and PNG = Highlanders of Papua New Guinea. The x-axis is years ago from modern times with the log scale. The y-axis is RCCR values calculated by Relate. The shaded regions are the 95 percent (two standard deviation) confidence intervals created from 10 separate random sampling events. The line represents the mean values. The dotted line represents the simulated data of best fitted model A.

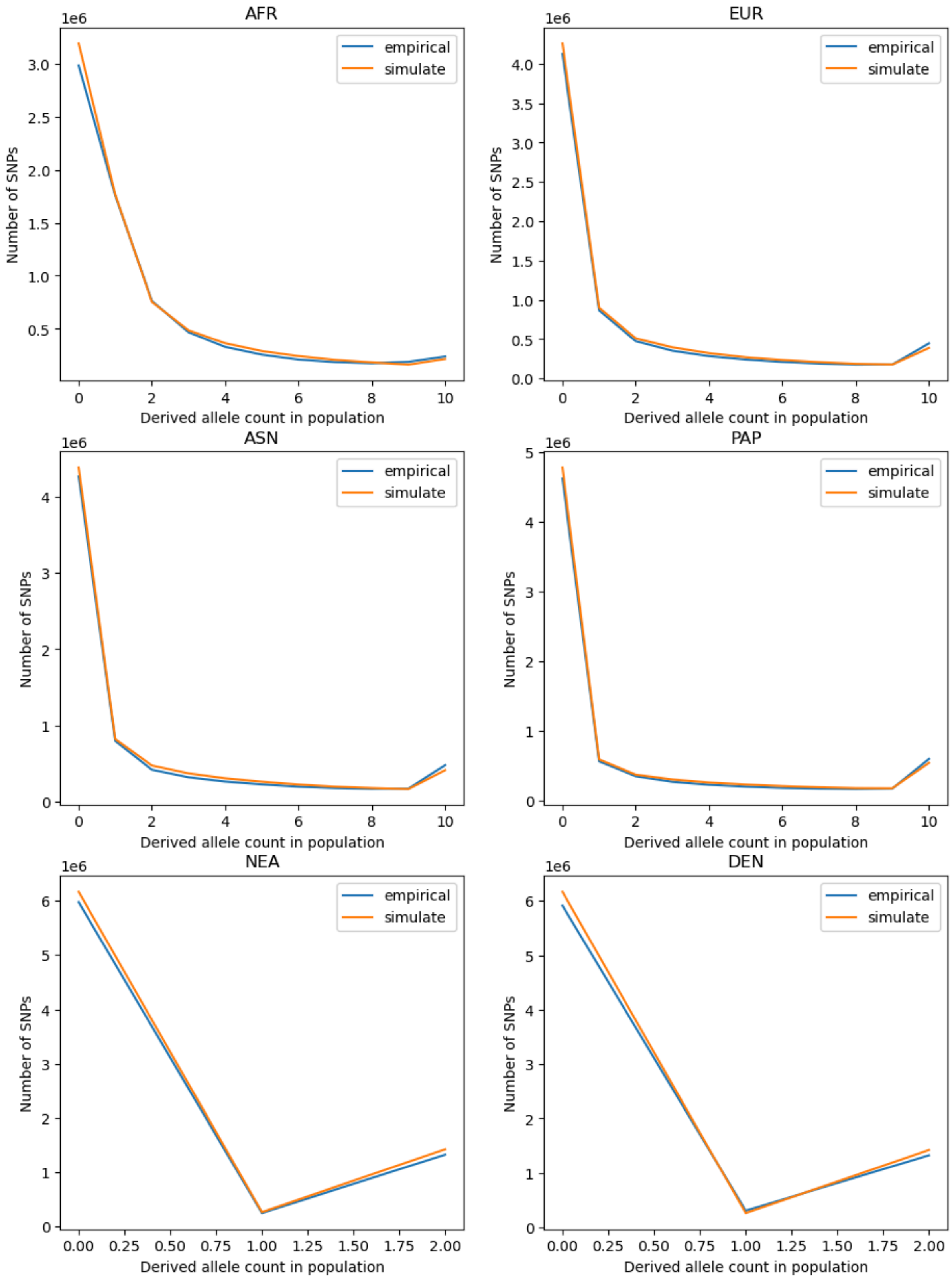
AFR
EUR
ASN
PAP
NEA
DEN
1e6
empirical
simulate
Number of SNPs
Derived allele count in population

Supplementary Figure 16: Site frequency spectrum comparison between empirical data and simulated data. AFR = Yoruba, EUR = British from England and Scotland, ASN = Han Chinese and PNG = Highlanders of Papua New Guinea, NEA= Altai Neanderthal, DEN=Denisova.

Supplementary Tables

Params	Model A	Model O	Model M	Model AX	Model OX
N_{A}	5,000 - 25,000	5,000 - 25,000	5,000 - 25,000	5,000 - 25,000	5,000 - 25,000
N_{AF}	10,000 - 60,000	10,000 - 60,000	10,000 - 60,000	10,000 - 60,000	10,000 - 60,000
N_{EU}	10,000 - 150,000	10,000 - 150,000	10,000 - 150,000	10,000 - 150,000	10,000 - 150,000
N_{AS}	10,000 - 150,000	10,000 - 150,000	10,000 - 150,000	10,000 - 150,000	10,000 - 150,000
N_{PA}	5,000 - 50,000	5,000 - 50,000	5,000 - 50,000	5,000 - 50,000	5,000 - 50,000
N_{NE}	500 - 10,000	500 - 10,000	500 - 10,000	500 - 10,000	500 - 10,000
N_{DE}	500 - 10,000	500 - 10,000	500 - 10,000	500 - 10,000	500 - 10,000
N_{EU0}	500 - 5,000	500 - 5,000	500 - 5,000	500 - 5,000	500 - 5,000
N_{AS0}	500 - 5,000	500 - 5,000	500 - 5,000	500 - 5,000	500 - 5,000
N_{PA0}	500 - 5,000	500 - 5,000	500 - 5,000	500 - 5,000	500 - 5,000
N_{B}	500 - 5,000	500 - 5,000	500 - 5,000	500 - 5,000	500 - 5,000
N_{ND}	500 - 30,000	500 - 30,000	500 - 30,000	500 - 30,000	500 - 30,000
T_{DPM} (ky)	10 - 100	10 - 100	10 - 100	10 - 100	10 - 100
T_{AS_PA} (ky)	0.5 - 50	NA	NA	NA	NA
T_{Mix} (ky)	NA	NA	0.5 - 50	10 - 100	0.5 - 50
T_{EU_AS} (ky)	0.5 - 50	10 - 100	0.5 - 50	0.5 - 50	10 - 100
T_{EA_PA} (ky)	NA	0.5 - 50	0.5 - 50	NA	0.5 - 50
T_{NOM} (ky)	0.5 - 50	0.5 - 50	0.5 - 50	0.5 - 50	0.5 - 50
T_{B} (ky)	0.5 - 50	0.5 - 50	0.5 - 50	0.5 - 50	0.5 - 50
T_{X_H} (ky)	NA	NA	NA	0.5 - 100	0.5 - 100
T_{AF} (ky)	0.5 - 200	0.5 - 200	0.5 - 200	0.5 - 200	0.5 - 200
T_{NI_NS} (ky)	10 - 500	10 - 500	10 - 500	10 - 500	10 - 500
T_{DI_DS} (ky)	10 - 500	10 - 500	10 - 500	10 - 500	10 - 500
T_{N_D} (ky)	10 - 500	10 - 500	10 - 500	10 - 500	10 - 500
T_{H_A} (ky)	100 - 500	100 - 500	100 - 500	100 - 500	100 - 500
${}^{NEA}m_{OOA}$ (%)	1 - 5	1 - 5	1 - 5	1 - 5	1 - 5
${}^{DEN}m_{PAP}$ (%)	1 - 5	1 - 5	1 - 5	1 - 5	1 - 5
Mix (%)	NA	NA	1 - 99	1 - 99	1 - 99

Supplementary Table 1: Prior range for different models; ky = thousand years

Simulation	A	O	M	AX	OX
A	84.89%	0.04%	12.33%	2.74%	0.00%
O	0.33%	81.97%	6.25%	0.42%	11.03%
M	13.95%	5.86%	71.86%	7.00%	1.33%
AX	6.21%	0.13%	10.37%	80.17%	3.12%
OX	0.00%	11.28%	0.78%	7.08%	80.87%
Empirical					
Proportion of accepted simulations (rejection)	94.96%	0.00%	1.93%	3.12%	0.00%
Goodness of fit p value	0.48	0	0.1	0.01	0

Supplementary Table 2: ABC-DLS classification results. First five rows are the mean misclassification probabilities of models calculated using the cross validation method using Approximate Bayesian Computation (ABC). The last two rows are calculated using empirical data. P value of Goodness of fit is calculated using ABC and is a one sided test. Here the null hypothesis is the data came from the corresponding statistical model.

Simulation	A	O	M	AX	OX
A	82.89%	0.30%	12.76%	4.03%	0.01%
O	0.07%	79.23%	7.55%	0.83%	12.33%
M	14.95%	5.96%	63.33%	13.46%	2.30%
AX	3.47%	1.30%	13.93%	78.55%	2.76%
OX	0.00%	10.49%	1.36%	3.84%	84.31%
Empirical					
Proportion of accepted simulations (rejection)	89.27%	0.00%	6.88%	3.85%	0.00%
Goodness of fit p value	0.99	0.0	0.17	0.0	0.07

Supplementary Table 3: ABC-DLS classification results with using stricter mask (without any genes with 10 kb flanking region) for empirical data. First five rows are the mean misclassification probabilities of models calculated using cross validation method. The last two rows are calculated using empirical data Approximate Bayesian Computation (ABC). P value of Goodness of fit is calculated using Approximate Bayesian Computation (ABC) and is a one sided test. Here the null hypothesis is the data came from the corresponding statistical model.

Simulation	A	O	M	AX	OX
A	87.34%	0.71%	7.95%	4.00%	0.00%
O	1.08%	87.41%	9.95%	1.48%	0.07%
M	9.51%	8.07%	62.38%	19.61%	0.44%
AX	2.12%	1.23%	22.24%	74.29%	0.12%
OX	0.00%	0.00%	0.00%	0.00%	100.00%
Empirical					
Proportion of accepted simulations (rejection)	91.21%	0.00%	6.27%	2.53%	0.00%
Goodness of fit p value	0.07	0	0.02	0	0

Supplementary Table 4: ABC-DLS results on classification with low migration rate ($m < 5 \times 10^{-5}$). First five rows are the mean misclassification probabilities of models calculated using cross validation method Approximate Bayesian Computation (ABC). The last two rows are calculated using empirical data. P value of Goodness of fit is calculated using ABC and is a one sided test. Here the null hypothesis is the data came from the corresponding statistical model.

Simulation	A	O	M	AX	OX
A	62.25%	16.28%	15.63%	5.81%	0.02%
O	14.69%	63.50%	12.08%	9.68%	0.05%
M	14.70%	10.96%	55.74%	18.59%	0.00%
AX	9.03%	8.01%	21.94%	61.01%	0.00%
OX	0.00%	0.00%	0.00%	0.00%	100.00%
Empirical					
Proportion of accepted simulations (rejection)	48.37%	44.33%	4.85%	2.45%	0.00%
Goodness of fit p value	0.24	0.5	0.16	0.25	0

Supplementary Table 5: ABC-DLS results on classification with high migration rate ($m < 5 \times 10^{-4}$). First five rows are the mean misclassification probabilities of models calculated using the cross validation method Approximate Bayesian Computation (ABC). The last two rows are calculated using empirical data. P value of Goodness of fit is calculated using ABC and is a one sided test. Here the null hypothesis is the data came from the corresponding statistical model.

Params	Description	Values
N_{AF}	Effective population size of modern African population	10,000
N_{EU0}	Effective population size of European population before exponential growth	10,000
r_{EU}	Increase of effective population size for Europeans per generation for exponential growth	0.2 %
N_{PA0}	Effective population size of PNG population before exponential growth	500-10,000
r_{PA}	Increase of effective population size for PNG per generation for exponential growth	0-0.2 %
T_{EU_PA}	Time event of separation between Europeans and PNG	50 kya
T_B	Time event of Separation of African and out of Africa population	80 kya

Supplementary Table 6: Parameter values used for a Minimal model of OOA

Params	Model A	Model O	Model M	Model AX	Model OX
**Denisova introgression to PNG**	T_{DPM}	T_{DPM}	T_{DPM}	T_{DPM}	T_{DPM}
**Separation between PNG and East Asian (A_P)**	$T_{DPM} + T_{AS\_PA}$	NA	NA	NA	NA
**Admixture between Asian and outgroup PNG (A_O)**	NA	NA	$T_{DPM} + T_{Mix}$	NA	NA
**Admixture between Asian and earlier OOA (A_X)**	NA	NA	NA	$T_{DPM}+ T_{Mix}$	NA
**Admixture between outgroup PNG and earlier OOA (O_X)**	NA	NA	NA	NA	$T_{DPM}+ T_{Mix}$
**Separation between European and Asian (E_A)**	A_P + T_{EU_AS}	T_{EU_AS}	A_O + T_{EU_AS}	AX + T_{EU_AS}	T_{EU_AS}
**Separation between Eurasian and outgroup PNG (EA_PA)**	NA	max (T_{DPM} + T_{EU_AS}) + T_{EA_PA}	E_A + T_{EA_PA}	NA	max (E_A, O_X) + T_{EA_PA}
**Neanderthal introgression to OOA population (NI)**	E_A + T_{NOM}	EA_PA + T_{NOM}	EA_PA + T_{NOM}	EA_PA + T_{NOM}	EA_PA + T_{NOM}
**Separation between**	NI + T_B	NI + T_B	NI + T_B	NI + T_B	NI + T_B

**OOA and African (OOA)**					
**Separation between earlier OOA and African (xOOA)**	NA	NA	NA	OOA + T_{X_H}	OOA + T_{X_H}
**Ancestral Size change**	OOA + T_AF	OOA + T_{AF}	OOA + T_{AF}	xOOA+ T_{AF}	xOOA+ T_{AF}
**Separation between Introgressed Neanderthal and Sequenced Neanderthal (NI_NS)**	NI + T_{NI_NS}	NI + T_{NI_NS}	NI + T_{NI_NS}	NI + T_{NI_NS}	NI + T_{NI_NS}
**Separation between Introgressed Denisova and Sequenced Denisova (DI_DS)**	T_{DPM} + T_{DI_DS}	T_{DPM} + T_{DI_DS}	T_{DPM} + T_{DI_DS}	T_{DPM} + T_{DI_DS}	T_{DPM} + T_{DI_DS}
**Split between Neanderthal Denisova (N_D)**	max(NI_NS, DI_DS) + T_{N_D}	max(NI_NS, DI_DS) + T_{N_D}	max(NI_NS, DI_DS) + T_{N_D}	max(NI_NS, DI_DS) + T_{N_D}	max(NI_NS, DI_DS) + T_{N_D}
**Split Between Human and Archaics**	max (N_D, OOA) + T_{H_A}	max (N_D, OOA) + T_{H_A}	max (N_D, OOA) + T_{H_A}	max (N_D, xOOA) + T_{H_A}	max (N_D, xOOA) + T_{H_A}

Supplementary Table 7: Relations of events with the time intervals used in the simulations
