# Supplementary material for: Resolving out of Africa event for Papua New Guinean population using neural network
Source: Nat Commun. 2025 Jul 9;16:6345. doi: 10.1038/s41467-025-61661-w (PMC12241555; doi:10.1038/s41467-025-61661-w)
Supplement: Supplementary file 2 — Description of Additional Supplementary Files [file 41467_2025_61661_MOESM2_ESM.pdf]

Description of Additional Supplementary Files

Supplementary Data: Information about the samples used in the manuscript.
