# Supplementary material for: Resolving out of Africa event for Papua New Guinean population using neural network
Source: Nat Commun. 2025 Jul 9;16:6345. doi: 10.1038/s41467-025-61661-w (PMC12241555; doi:10.1038/s41467-025-61661-w)
Supplement: Supplementary file 4 — Reporting Summary [file 41467_2025_61661_MOESM4_ESM.pdf]

nature portfolio

Corresponding author(s): Mayukh Mondal

Last updated by author(s): Jun 10, 2025

Reporting Summary

Nature Portfolio wishes to improve the reproducibility of the work that we publish. This form provides structure for consistency and transparency in reporting. For further information on Nature Portfolio policies, see our Editorial Policies and the Editorial Policy Checklist.

Statistics

For all statistical analyses, confirm that the following items are present in the figure legend, table legend, main text, or Methods section.

n/a	Confirmed	
☐	☒	The exact sample size (*n*) for each experimental group/condition, given as a discrete number and unit of measurement
☐	☒	A statement on whether measurements were taken from distinct samples or whether the same sample was measured repeatedly
☐	☒	The statistical test(s) used AND whether they are one- or two-sided<br>*Only common tests should be described solely by name; describe more complex techniques in the Methods section.*
☒	☐	A description of all covariates tested
☒	☐	A description of any assumptions or corrections, such as tests of normality and adjustment for multiple comparisons
☐	☒	A full description of the statistical parameters including central tendency (e.g. means) or other basic estimates (e.g. regression coefficient) AND variation (e.g. standard deviation) or associated estimates of uncertainty (e.g. confidence intervals)
☒	☐	For null hypothesis testing, the test statistic (e.g. *F*, *t*, *r*) with confidence intervals, effect sizes, degrees of freedom and *P* value noted<br>*Give P values as exact values whenever suitable.*
☐	☒	For Bayesian analysis, information on the choice of priors and Markov chain Monte Carlo settings
☒	☐	For hierarchical and complex designs, identification of the appropriate level for tests and full reporting of outcomes
☒	☐	Estimates of effect sizes (e.g. Cohen's *d*, Pearson's *r*), indicating how they were calculated

Our web collection on statistics for biologists contains articles on many of the points above.

Software and code

Policy information about availability of computer code

Data collection	No new data was collected.
Data analysis	ABC-DLS v2.1.2<br>png_xOOA v1.0<br>Relate v1.1.8<br>SHAPEIT v4.2<br>BWA-MEM v0.7.12<br>GATK v4.2.0<br>bcftools v1.9<br>samtools v1.9

For manuscripts utilizing custom algorithms or software that are central to the research but not yet described in published literature, software must be made available to editors and reviewers. We strongly encourage code deposition in a community repository (e.g. GitHub). See the Nature Portfolio guidelines for submitting code & software for further information.

Data

Policy information about availability of data

All manuscripts must include a data availability statement. This statement should provide the following information, where applicable:

- Accession codes, unique identifiers, or web links for publicly available datasets
- A description of any restrictions on data availability
- For clinical datasets or third party data, please ensure that the statement adheres to our policy

No data was generated for this manuscript. We used PNG data available in EGA under accession number EGAD00001010142, EGAD00001010143 and EGAD50000000050. The 1000 genome data was downloaded from the specific website (https://www.internationalgenome.org/data-portal/data-collection/30x-grch38). The Andamanese data set is downloaded from ENA with accession number PRJEB11455. Neranderthal genome was downloaded from http://cdna.eva.mpg.de/neandertal/altai/AltaiNeandertal/VCF/ and Denisova genome was downloaded from http://cdna.eva.mpg.de/denisova/VCF/. Ancestral fasta file is downloaded from ensemble website (https://ftp.ensembl.org/pub/release-105/fasta/ancestral_alleles/homo_sapiens_ancestor_GRCh38.tar.gz). The GRCh38 genome reference was built into the GATK workflow (https://console.cloud.google.com/storage/browser/genomics-public-data/resources/broad/hg38/v0/). The source data for the figures and empirical cSFS generated in this manuscript can be found in figshare repository under 29222954 (https://doi.org/10.6084/m9.figshare.29222954).

Research involving human participants, their data, or biological material

Policy information about studies with human participants or human data. See also policy information about sex, gender (identity/presentation), and sexual orientation and race, ethnicity and racism.

Reporting on sex and gender	All the analysis is done using random sampling on publicly available dataset. Thus our results work equally for both sexes.
Reporting on race, ethnicity, or other socially relevant groupings	We did not include or exclude samples from out analysis based on race, ethnicity or other socially relevant groupings.
Population characteristics	We used publicly available data. We used the major well known populations from 1000 genome data (Yoruba, Han Chinese South and British from England and Scotland) and we chose the Papuan New Guinean data published in our previous work.
Recruitment	We did not recruit the samples. We randomly sampled individuals from all the samples available from publicly available data.
Ethics oversight	University of Tartu

Note that full information on the approval of the study protocol must also be provided in the manuscript.

Field-specific reporting

Please select the one below that is the best fit for your research. If you are not sure, read the appropriate sections before making your selection.

☒ Life sciences ☐ Behavioural & social sciences ☐ Ecological, evolutionary & environmental sciences

For a reference copy of the document with all sections, see nature.com/documents/nr-reporting-summary-flat.pdf

Life sciences study design

All studies must disclose on these points even when the disclosure is negative.

Sample size	We are bound by the sample size coming from publicly available data. In the case of the ABC-DLS and Relate we can reach significance with such numbers.
Data exclusions	No further exclusion was done on this manuscript. We kept the exclusion principle presented from the source of data.
Replication	Due to the nature of the data (high coverage PNG data), our analysis cannot be directly replicated right now due to availability of data. Nonetheless, we reproduced our RELATE results with a smaller sample size coming from the HGDP dataset (mentioned in the manuscript).
Randomization	Instead of using replication, we used randomization and subsampled the data. Thus we have replicated our data in multiple randomly subsampled data.
Blinding	Not applicable as we did not collect the data. When subsampled we used a random generator thus essentially blinded.

Reporting for specific materials, systems and methods

We require information from authors about some types of materials, experimental systems and methods used in many studies. Here, indicate whether each material, system or method listed is relevant to your study. If you are not sure if a list item applies to your research, read the appropriate section before selecting a response.

Materials & experimental systems

n/a	Involved in the study
☒	☐ Antibodies
☒	☐ Eukaryotic cell lines
☒	☐ Palaeontology and archaeology
☒	☐ Animals and other organisms
☒	☐ Clinical data
☒	☐ Dual use research of concern
☒	☐ Plants

Methods

n/a	Involved in the study
☒	☐ ChIP-seq
☒	☐ Flow cytometry
☒	☐ MRI-based neuroimaging

Plants

Seed stocks

Report on the source of all seed stocks or other plant material used. If applicable, state the seed stock centre and catalogue number. If plant specimens were collected from the field, describe the collection location, date and sampling procedures.

Novel plant genotypes

Describe the methods by which all novel plant genotypes were produced. This includes those generated by transgenic approaches, gene editing, chemical/radiation-based mutagenesis and hybridization. For transgenic lines, describe the transformation method, the number of independent lines analyzed and the generation upon which experiments were performed. For gene-edited lines, describe the editor used, the endogenous sequence targeted for editing, the targeting guide RNA sequence (if applicable) and how the editor was applied.

Authentication

Describe any authentication procedures for each seed stock used or novel genotype generated. Describe any experiments used to assess the effect of a mutation and, where applicable, how potential secondary effects (e.g. second site T-DNA insertions, mosiacism, off-target gene editing) were examined.
